# Effect of dental tissue thickness on the measurement of oxygen saturation by two different pulse oximeters

**DOI:** 10.1590/0103-6440202204903

**Published:** 2022-10-21

**Authors:** Dilma Helena Neves Henriques, Ana Maria Hecke Alves, Morgane Marion Kuntze, Lucas da Fonseca Roberti Garcia, Eduardo Antunes Bortoluzzi, Cleonice da Silveira Teixeira

**Affiliations:** 1 Department of Dentistry, Federal University of Santa Catarina, Florianópolis, Santa Catarina, Brazil.; 2 Department of Diagnosis & Oral Health; Division of Endodontics, School of Dentistry, University of Louisville, KY, USA

**Keywords:** dental pulp test, dental tissues, oxygen saturation, photoplethysmography, pulse oximetry

## Abstract

This study aimed to evaluate the influence of different dental tissue thickness on the measurement of oxygen saturation (SpO_2_) levels in high (HP) and low (LP) blood perfusion by comparing the values obtained from two different pulse oximeters (POs) - BCI and Sense 10. Thirty freshly extracted human teeth had their crowns interposed between the POs and an optical simulator, which emulated the SpO_2_ and heart beats per minute (bpm) at HP (100% SpO_2_/75 bpm) and LP (86% SpO_2_/75 bpm) modes. Afterwards, the palatine/lingual surfaces of the dental crowns were worn with diamond drills. The reading of SpO_2_ was performed again using the POs alternately through the buccal surface of each dental crown. Data were analyzed by the Wilcoxon, Mann-Whitney and Kendall Tau-b tests (α=5%). The results showed significant difference at the HP and LP modes in the SpO_2_ readouts through the different dental thicknesses with the use of BCI, and at the LP mode with the use of Sense 10, which had a significant linear correlation (p<0.0001) and lower SpO_2_ readout values in relation to the increase of the dental thickness. Irrespective of tooth thickness, Sense 10 had significantly higher readout values (p<0.0001) than BCI at both perfusion modes. The interposition of different thicknesses of enamel and dentin influenced the POs measurement of SpO_2,_ specially at the low perfusion mode. The POs were more accurate in SpO_2_ measurement when simulated perfusion levels were higher.

## Resumo

Este estudo avaliou a influência de diferentes espessuras de esmalte e dentina na medição dos níveis de saturação de oxigênio (SpO_2_) em alta (HP) e baixa (LP) perfusão sanguínea, comparando os valores obtidos em dois oxímetros de pulso (OPs) diferentes, BCI e Sense 10. Trinta dentes recém-extraídos de humanos tiveram suas coroas interpostas entre os OPs e um simulador óptico, que simulava a SpO_2_ e os batimentos cardíacos por minuto (bpm) nos modos de HP (100% SpO_2_ / 75 bpm) e LP (86% SpO_2_ / 75 bpm). Após, as superfícies palatinas / linguais dos dentes foram desgastadas com brocas de diamantadas. A leitura da SpO_2_ foi realizada novamente usando os dois OPs alternadamente através da face vestibular de cada coroa dental. Os dados foram analisados ​​pelos testes Wilcoxon, Mann-Whitney e Kendall Tau-b (α = 5%). Os resultados mostraram diferença significativa nos modos HP e LP nas leituras de SpO_2_ através das diferentes espessuras dentárias com o uso do BCI, e no modo LP com o uso do Sense 10, que teve correlação linear significativa (p <0,0001) e menores valores de leitura de SpO_2_ em relação ao aumento da espessura dentária. Independentemente da espessura do dente, o Sense 10 apresentou valores de leitura significativamente maiores (p <0,0001) do que o BCI em ambos os modos de perfusão. A interposição de diferentes espessuras de esmalte e dentina influenciaram a mensuração da SpO_2_ pelos OPs, especialmente no modo de baixa perfusão. Os POs foram mais precisos na mensuração da SpO_2_ quando os níveis simulados de perfusão foram maiores.

## Introduction

The Pulse Oximeter (PO) is a device widely used in medicine for monitoring the vital functions of patients ^1^. In the last years, the PO has been used as an objective, atraumatic and non-invasive alternative for assessing dental pulp vitality ^2^ ^,^ ^3^ ^,^ ^4^ ^,^ ^5^. POs operate based on the measurements of different absorptions of red and infrared light from oxyhemoglobin and deoxyhemoglobin, by showing pulse rate and the blood oxygen saturation (SpO_2_) of the tissues ^6^.

The pulse oximetry is based on two principles: spectrophotometry, which determines the concentration of a substance in a solution, by measurement of the quantity of light transmitted or reflected; and plethysmography, the capability of measuring the variations of ongoing volume of blood pulsation in different extremities of the body (ear, toes, fingers and nose) ^1^. Due to these characteristics, and the fact that PO detects pulp vitality by the preSense of pulpal blood flow, the use of oximeter does not promote painful responses ^7^. Therefore, it is more advantageous than other tests that are used for tooth sensibility detection, such as the thermal (heat and cold stimuli) and electric types, in addition of being more accepted by patients ^8^.

Although PO was not originally developed for SpO_2_ of dental pulp measuring, several laboratory and clinical studies have been conducted with the purpose of inserting this tool as a method of diagnosis of pulp vitality in endodontics ^2^ ^,^ ^3^ ^,^ ^4^ ^,^ ^5^ ^,^ ^7^ ^,^ ^9^ ^,^ ^10^. Scientific findings have shown lower mean values of SpO_2_ of dental pulp in comparison with the mean SpO_2_ values measured in the fingers of patients ^4^ ^,^ ^7^ ^,^ ^11^. A plausible justification relies on the preSense of enamel and dentin structures, which hinders the detection of pulp vascularization ^12^. Furthermore, the variation in the thicknesses of enamel and dentin would explain the difference among the mean SpO_2_ values found in anatomically distinct groups of teeth, such as maxillary central incisors (91.29%) ^7^, maxillary canines (90.69%) ^11^; maxillary premolars (86.2%) ^4^; maxillary molars (83.59%) and mandibular molars (86.89%) ^5^. Some of these studies included negative control groups with endodontically treated teeth obtaining mean SpO_2_ values of 0% ^9^ ^,^ ^11^, and groups of teeth with necrotic pulp, with mean SpO_2_ values of 74.6% ^9^.

In view of these scientific results, new studies are necessary to consolidate the previously published findings. Therefore, the aim of this study was to evaluate the effect of dental tissue thickness on the measurement of SpO_2_, simulating high (HP) and low (LP) blood perfusion modes, and to compare the readout values obtained by two different POs, BCI and Sense 10. Two null hypotheses were formulated: 1) the dental structures (enamel and dentin) would not interfere in the measurement of the SpO_2_, regardless of the perfusion mode evaluated; 2) the two oximeters (BCI and Sense 10) tested would not differ in the measurement of the SpO_2_ levels, regardless of the perfusion mode or the dental tissue thickness.

## Materials and methods

### Sample size calculation

The sample size was estimated based on studies that evaluated the SpO_2_ in 67 anterior teeth ^13^, 17 maxillary incisors ^14^ and 80 canines and premolars ^15^. Therefore, with α = 0.05 and a power of 80%, the analysis of 30 teeth was required to perform the experiment.

### Selection of teeth and preparation of specimens

After approval from the Ethics Committee on Human Research of the Federal University of Santa Catarina (Protocol No 2.345.915), 30 non-carious human maxillary/mandibular incisors and canines, extracted for reasons not related to this study, were selected. Initial periapical radiographs were taken with the aid of the Spectro 70X Selectronic X-ray machine (Dabi Atlante, Ribeirão Preto, SP, Brazil) using a size 2 digital sensor (STD 900201, Imaging Plates, Soredex, Tuusula, Finland). The exposure parameters were 70 kV, 0.8 mA and 0.25 s. The inclusion criteria required permanent teeth free of caries, restorations and intrapulp calcification. Teeth with visible changes in their crowns were excluded from the sample. After the calculus and remaining soft tissues removal, the teeth were stored in 0.1 % thymol solution at 4 ^o^C, and used within 3 months after extraction.

The crown thickness of each tooth was measured in the buccal-palatine/lingual direction at the middle third of the crown, by using a Mitutoyo absolute digital caliper (Mitutoyo, Kanogawa, Japan) with an accuracy of 0.001 mm. The mean (standard deviation) thickness of the crowns was 5.01 (± 1.05) mm, ranging from 4.0 mm to 6.8 mm.

### SpO_2_ measurement through dental crowns

**Figure. 1:**
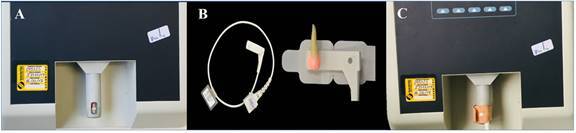
(A) Illustrative image showing an optical ‘finger’ (Simulator). (B) The sensor 3025 (Smiths Medical) was used to envolve each tooth. (C) The sensor with the tooth adapted surrounded the ‘finger’ at the optical Simulator, with the light emitter diode (red LED) positioned in the lower part, and the receiver diode in the upper part of the device.

To perform the SpO_2_ readout through the dental crowns, a simulator with an optical ‘finger’ (Simulator) (Fluke Index 2XL, Fluke Biomedical, Everett, Washington, USA) was used to emulate the oxygen saturation patterns and heart rate per minute (Figure 1A). To ensure a proper SpO_2_ readout, the simulator was submitted to calibration right before its use in the experiment. The two POs used in this study, namely SENSE 10 (Alfamed Medical Systems, Curitiba, Paraná, Brazil) and BCI (Smiths Medical PM Inc., Waukesha, WI, USA), were also previously tested regarding their precision by the IEB Laboratory (Institute of Biomedical Engineering).

The measurements were performed by only one operator, in two different modes: simulation of the parameters at high perfusion (HP) or low perfusion (LP). Initially, the simulator was programmed for HP levels, with parameters set at 100% of SpO_2_ and 75 beats per minute (bpm). Subsequently, the LP levels were simulated at 86% of SpO_2_ and 75 bpm.

The SpO_2_ measurements were taken by interchanging the use of both POs. The 3025 sensor (Smiths Medical) (Figure 1B) was used for the simulator.

The first measurement was obtained individually by each device without the tooth and represented the positive control (reference values). The sensor 3025 was positioned surrounded the ‘finger’ at the optical simulator. The light emitter diode (red LED) was positioned in the lower part, and the receiver diode in the upper part of the device (Figure 1C). In the sequence, the second measurement was taken with each dental crown properly positioned under the red LED of the sensor 3025, alternating the POs (Sense 10 and BCI), as described for the positive control. Extreme care was taken to ensure that the red LED and the dental crown were positioned at the bottom, and the receiving diode, at the top of the optical ‘finger’, in the area relative to the pulp chamber of the tooth, so that they were parallel. For this, during each reading, a box with a black background was used over the entire apparatus (simulator, tooth and sensor) to prevent the exposure to ambient light.

### SpO_2_ measurement through worned dental crowns

After the measurement of the SpO_2_ through the crowns, the palatine/lingual surfaces of each dental crown was worn with a conical diamond bur (4138, KG Sorensen, Cotia, SP, Brazil), mounted at high-speed device, under copious air/water cooling spray, in order to reduce approximately 2 mm from its original thickness. The crown thickness of each tooth was again measured (Mitutoyo), and the mean (standard deviation) thickness of the crowns after worn was 2.92 (0.79) mm, ranging from 2.0 mm to 4.9 mm. Next, the SpO_2_ readout through the worn out crown was performed, as described for the entire crown, using the optical simulator set at the HP (100% SpO_2_, 75 bpm) and LP (86% SpO_2_, 75 bpm) modes. The same POs used previously were checked with the use of the same sensor 3025, coupled to the optical simulator, as described before. In the same way, one initial measure without a dental crown coupled to the simulator was used as positive control of each POs. The readout values were taken and tabulated with respect to the maximum time of 30 seconds (time for stabilization of the values during the SpO_2_ measurement).

### Statistical analysis

The statistical analysis was performed with the SPSS Statistics 21 software (SPSS Inc., Chicago, IL, EUA), with significance level set at 5%. The normality of the data was evaluated by Kolmogorov-Smirnov and Shapiro-Wilk tests. As the sample did not present normal distribution (p<0.0001), the data were analyzed by the non-parametric Wilcoxon, Kendall rank correlation coefficient (Kendall Tau-*b*) and Mann-Whitney tests.

## Results

**Figure. 2:**
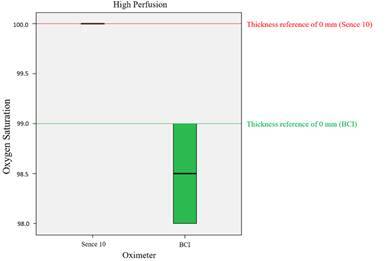
Comparison between the SpO_2_ levels measured through different tooth structure thicknesses (2.1 to 6.8 mm) with BCI and Sense 10 in HP mode.

**Figure. 3:**
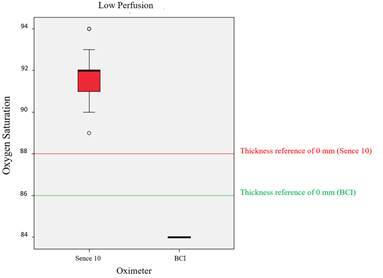
Comparison between the SpO_2_ levels measured through different tooth structure of thickness (2.1 to 6.8 mm) with BCI and Sense 10 in LP mode.

**Figure. 4:**
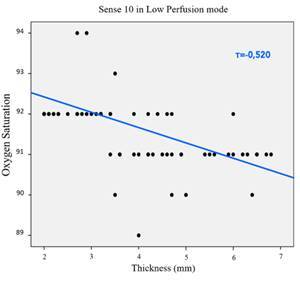
Correlation between the thickness (2.1 to 6.8 mm) and the SpO_2_ levels measured with Sense 10 in LP mode.

Figures 2 to 4 and Tables 1 and 2 demonstrate the results of the present study. Figures 2 (HP mode) and 3 (LP mode) show the SpO_2_ levels measured by the different POs through the different tooth structure thicknesses (from 2.1 to 6.8 mm), in comparison with the SpO_2_ reference values (measured directly without the interposition of tooth structures; thickness 0.0 mm).

The reference values (SpO_2_ reading of each PO without tooth interposition) considered for the combinations of PO and modes (LP and HP) were: BCI at LP mode: 86%, BCI at HP mode: 99%, Sense 10 at LP mode: 88%, and Sense 10 at HP mode: 100%. A significant difference was found (Wilcoxon, Mann-Whitney, p<0.0001) among the median (BCI at LP mode-84%, BCI at HP mode-98.5% and Sense 10 at LP mode-92%) and the reference values for all combinations, except for Sense 10 (median of 100%) at HP mode (p=1.0). Therefore, interposition of the tooth structures significantly changed the SpO_2_ readout level measured with BCI at both modes, and Sense 10 Alfamed at LP mode.

The correlation between the thickness (2.1-6.8 mm) and the SpO_2_ levels, measured with the two POs at both modes, demonstrated that only Sense 10 at LP mode had significant linear correlation (tau*-b* of Kendall, p<0.0001, τ_β_=-0.520, Figure 4). In other words, the SpO_2_ measurement levels showed a trend towards diminishing with the increase of tooth structure thickness. No significant linear correlation was found (p>0.05) for the remaining PO/mode combinations.

The comparison between the SpO_2_ levels obtained with the two POs set at both LP and HP modes, irrespective of the overlapping tooth structure thickness, showed significant difference (Wilcoxon, Mann-Whitney, p<0.0001) between the devices. The SpO_2_ values measured with Sense 10 at both LP and HP modes were significantly higher than the values measured with BCI at both modes.

**Table 1:** Medians (first / third quartiles) and standard deviation of oxygen saturation levels measured at the different thickness of enamel and dentin structures for the different dental groups (intact tooth crown).*

Dental Groups ^ϯ^ (Intact tooth crowns) Pulse Oximeter	UCI + ULI (n=11) Thickness 4.6mm (4.1mm/4.7mm) 0.39		UC + LC(n=12) Thickness 6.2mm (5.9mm/6.5mm) 0.5		LCI (n=7) Thickness 3.8mm (3.6mm/4.2mm) 0.41	
	BCI	Sense 10	BCI	Sense 10	BCI	Sense 10
LP With/Tooth** (86% SaO2)	84(84/84)0^B,b^	91(91/92)0.94^A,a^	84(84/84)0^B,b^	91(91/91) 0.41^A,a^	84(84/84) 0^B,b^	91(91/91) 0.51^A,a^
LP Without/Tooth (86% SaO2)	86(86/86)0 ^B,a^	88(88/88)0^A b^	86(86/86)0^B,a^	88(88/88)0^A,b^	86(86/86) 0^B,a^	88(88/88) 0 ^A,b^
HP With/Tooth (100% SaO2)	99(99/99)0.40^B,a^	100(100/100)0^A, a^	98(98/99)0.48^B,b^	100(100/100)0.60^A,a^	98(98/99)0.52^B,a^	100(100/100) 0 ^A,a^
HP Without/Tooth (100% SaO2)	99(99/99)0 ^B, a^	100(100/100)0 ^A, a^	99(99/99)0 ^B, a^	100(100/100)0 ^A, a^	99(99/99) 0 ^B, a^	100(100/100)0 ^A, a^

*Medians accompanied by the same superscript capitalized letters in the same line did not show significant difference (Mann-Whitney U, p> 0.05). Medians accompanied by the same superscript lower-case letters in the same column (at LP, or at HP mode) did not show significant difference (Mann-Whitney U, p> 0.05).

**The Sense 10 PO presented outliner values (higher than 2%) for the measurement performed at LP mode, with the interposition of the tooth (enamel and dentin structures). LP: Low blood perfusion; HP: High blood perfusion; PO (pulse oximeter).

^ϯ^ upper central incisor + upper lateral incisor ((UCI + ULI); lower central incisor (LCI); upper canine + lower canine (UC + LC). LP (lower perfusion mode); HP (high perfusion mode);

**Table 2:** Medians (first / third quartiles) and standard deviation of oxygen saturation levels measured at the different thickness of enamel and dentin structures for the different dental groups (worn tooth crown).*

Dental Groups ^ϯ^ (Worn tooth crowns) Pulse Oximeter	UCI + ULI (n=11) Thickness 2mm (2mm/2mm) 0		UC + LC(n=12) Thickness 3mm (3mm/3mm) 0		LCI (n=7) Thickness 2mm (2mm/2mm) 0	
	BCI	Sense 10	BCI	Sense 10	BCI	Sense 10
LP With/Tooth** (86% SaO2)	84(84/84)0^B,b^	92(92/92)0^A,a^	84(84/84)0^B,b^	92(92/92)0^A, a^	84(84/84)0^B,b^	92(92/92)0.41^A,a^
LP Without/Tooth (86% SaO2)	86(86/86) 0^B,a^	88(88/88)0^A, b^	86(86/86)0^B, a^	88(88/88)0^A, b^	86(86/86)0^B, a^	88(88/88)0 ^A, b^
HP With/Tooth (100% SaO2)	99(99/99)0.45^A,a^	10 (100/100)0 ^A,a^	98(98/98)0.46^B,b^	100(100/100)0.60^A,a^	98(98/99)0.49^B,b^	100(100/100)0^A,a^
HP Without/Tooth (100% SaO2)	99(99/99)0^B, a^	100(100/100)0^A,a^	99(99/99)0^B, a^	100(100/100)0 ^A, a^	99(99/99)0^B, a^	100(100/100)0^A,a^

*Medians accompanied by the same superscript capitalized letters in the same line did not show significant difference (Mann-Whitney U, p> 0.05). Medians accompanied by the same superscript lower-case letters in the same column (at LP, or at HP mode) did not show significant difference (Mann-Whitney U, p> 0.05).

**The Sense 10 PO presented outliner values (higher than 2%) for the measurement performed at LP mode, with the interposition of the tooth (enamel and dentin structures). LP: Low blood perfusion; HP: High blood perfusion; PO (pulse oximeter).

ϯ upper central incisor + upper lateral incisor ((UCI + ULI); lower central incisor (LCI); upper canine + lower canine (UC + LC). LP (lower perfusion mode); HP (high perfusion mode).

## Discussion

The results obtained in this study led to the rejection of both null hypothesis initially formulated. The thickness of the tooth structures (enamel and dentin) interfered in the SpO_2_ levels measurement. Moreover, the results showed significant difference between the HP and LP modes in SpO_2_ readout through the different dental thicknesses with the use of BCI, and at the LP mode with the use of Sense 10. In addition, Sense 10 presented significant linear correlation and lower SpO_2_ values in relation to the increase in tooth structure thickness. When the two POs were compared independently of tooth structure thickness, the Sense 10 obtained significantly higher SpO_2_ readout values than the BCI device, at both perfusion modes. These results could be explained mainly by the differences in performance and in functionality of the POs, as well as the variation in tooth structure thickness.

In order to understand the performance of the POs used in this study, it is necessary to highlight some aspects from the methodology employed. The simulator used in this study was the Fluke Index 2XL (Fluke Biomedical), and it is able to simulate the human finger, as well as heartbeat and the oxygen saturation levels between 35% and 100%, with 1% resolution ^16^. This device is used in repairing services, verification and calibration of functional performance of POs ^16^. In the present study, before the experiment began, the simulator's calibration capability was carefully verified.

POs are calibrated during their fabrication, and when turned on, their internal circuits are checked immediately. A pre-evaluation showed that these devices had greater precision in the measurement of SpO_2_ values, from 80% to 100%, with an acceptable range of errors of 2%, and inaccurate SpO_2_ readout for values of less than 80% ^17^. Other authors have considered that the precision of the PO readout tends to get worse when the SpO_2_ is lower than 90% ^1^ ^,^ ^16^. For this reason, in this study, the SpO_2_ values over 80% were chosen in both simulations, LP (86%/75) and HP (100%/75), and for both POs used.

The BCI 3301 presented median SpO_2_ values of 98.5% at HP and 84% at LP. Therefore, BCI functioned correctly in the acceptable range of error. Conversely, Sense 10 showed median SpO_2_ values of 100% at HP mode, and 92% at LP mode, which confirmed its functioning within a range of more or less 2% only in HP. In LP mode, this PO remained outside of the range of acceptable error ^17^.

Regarding the functionality of the POs, the conventional PO had some significant limitations caused by patient movements (which may cause displacement of the sensor) and by the low peripheral perfusion found in some clinical situations ^1^ ^,^ ^6^ ^,^ ^16^. Many types of POs functionality depends on the satisfactory arterial perfusion of the tissues ^1^. LP situations, as observed in cases of vessel constriction, hypothermia and hypoxia, may hinder the sensor in distinguishing between true signals and noises (undesirable signals), causing false results ^6^. The PO Sense 10 has high sensitivity and improved technology, as it was developed with the purpose of being used at lower perfusion mode ^6^ ^,^ ^18^. The greater sensitivity of this PO might explain the highest SpO_2_ levels readout at LP mode, when compared with the BCI device used in this study. However, it is important to point out that the overestimation of SpO_2_ may be harmful to patient monitoring, as it may cause delay in institution of treatment measures ^16^. In a recent laboratory study ^16^, several POs were tested using the same simulator (Fluke Index 2XL) used in the present study. The authors concluded that the accuracy was reduced at LP mode, with some POs showing SpO_2_ redout values much higher than those simulated by the simulator ^16^

In addition to the clinical limitations, oximetry also presented technical restrictions that may affect the SpO_2_ and bpm readouts obtained by POs ^19^. Some of these technical restrictions are electronic interferences, calibration process, defects, such as penumbra (caused by bad positioning of the sensor), interference of other sources of light and dependence on the pulse perception ^1^. It is valid to emphasize that these clinical and technical limitations are better controlled in a laboratory environment, which explains the higher values obtained in the SpO_2_ measurement in the present study, in comparison with other *in vivo* studies ^4^ ^,^ ^5^.

Concerning the variation in tooth structure thickness, the different thicknesses influenced the readouts performed by both POs, although only the data obtained with Sense 10, at LP mode, presented significant linear correlation with the dental thickness. These findings corroborate with a recently published study, which reported that the thickness of the dental structures interfere in the SpO_2_, both in the preSense and in the abSense of ambient light, with lower levels of SpO_2_ in teeth with greater thickness ^20^. However, it is worth mentioning that in the present study, in addition to evaluating the influence of thickness on SpO_2_, the performance and precision of two different POs were also evaluated, using an optical finger simulator, simulating two different parameters of perfusion (LP and HP). Our methodology differs in several other points from the methodology used by the studies mentioned above, who found that in the preSense of ambient light an average SpO_2_ of 96.3% and bpm of 69.5 was found and in the abSense of ambient light the average was 96% and bpm was 70.5 ^20^. Our study, in turn, taking into account the previously described parameters, found higher SpO_2_ redout values. Further, we demonstrated that the accuracy of the POs was shown to be greater, when levels at HP mode were simulated. The difference in the values ​​found in the two studies, may be explained by the differences in their study design, such as the group of teeth used, the enamel/dentin thickness established by each study, the use of different POs and sensors and, perhaps, one of the most important points, the fact that we used an optical finger simulator in the readings and the previous study used the patient's finger. In addition, we used a black box around the PO sensor when it was attached to the optical finger, in order to reduce the interference of ambient light.

Other clinical studies have shown that the SpO_2_ levels obtained through tooth structures appeared to be lower, when compared with the SpO_2_ values measured on the patients’ fingers ^4^ ^,^ ^7^ ^,^ ^11^ ^,^ ^21^. Some justifications may explain this difference. The preSense of enamel and dentin hinders the proper obtainment of SpO_2_ in the tooth ^7^, as the pulp is completely covered by these structures, which overlap and compromise the identification of vascularization ^2^ ^,^ ^7^ ^,^ ^12^. Another possibility is that the diffraction of infra-red light through enamel prisms and dental tubules is capable of detecting inaccurate levels of low SpO_2_ ^22^.

Several studies have shown lower SpO_2_ levels for the BCI device, and other different PO models, than those obtained in the present study ^7^ ^,^ ^10^ ^,^ ^21^. An *in vivo* study used the PO Criticare 504-US model with one clip-type sensor, and showed SpO_2_p mean levels of 85.11% for maxillary central incisors; 80.21% for maxillary lateral incisors; 89.55% for maxillary canines, and 95.88% for the finger (control group) ^10^. The BCI device, in a set with the sensor 3025, was also used in another *in vivo* study and registered SpO_2_ mean levels of 85.27% in the tooth, and SpO_2_ of 92.85% in the fingers ^7^. Another study showed SpO_2_ mean levels of 81.25% ± 8.19% in the tooth, and observed SpO_2_ of 95.77% ± 2 in the fingers ^21^.

Differently from the majority of studies published in the literature, this research was conducted *ex vivo*, which probably also contributed to the highest SpO_2_ readout values obtained by both devices, when compared with the previously cited studies ^2^ ^,^ ^7^ ^,^ ^10^ ^,^ ^21^. Moreover, standardization of the tests, by the use of the PO tester with the optical finger, diminished the variables and limitations of the PO observed *in vivo* (movement of the patients, variations of individual blood perfusion and electrical interference) ^6^.

Pulse oximetry has been used quite frequently in Dentistry clinical situations, mainly because it is a method that assesses the vascular condition of the tooth and provides a direct, objective and reliable method for testing pulp vitality of traumatized teeth ^15^ ^,^ ^23^ and in teeth of young patients ^7^ ^,^ ^24^. In addition, pulse oximetry has other attributes such as not being an invasive method, not causing pain, easy to apply and, among the vitality tests, being less costly ^8^ ^,^ ^21^ ^,^ ^25^. Other studies have shown that PO can help to determine the different inflammatory stages of the dental pulp ^9^ and in the diagnosis of different pulp pathologies, especially in the preSense of pulp necrosis, which can be inadequately interpreted by thermal tests ^25^.

However, pulse oximetry devices still need to be better developed for use in dentistry ^9^. Our results showed that, although Sense 10 was submitted and approved in the calibration test carried out prior to the study, its functionality was affected when it was used in LP mode, which resulted in differences greater than 2% in SaO_2_ readings. These results suggest that differences in the function and sensitivity among POs may cause different results to be observed in the studies, according to the type of device and sensor being used. Therefore, it is important that other laboratory and clinical studies are developed so that such doubts are resolved.

In conclusion, the results of the present study demonstrated that the interposition of different thicknesses of enamel and dentin influenced the POs measurement of SpO_2_, specially at LP mode. Increased enamel and dentin thickness promoted a decrease in the SpO_2_ readout values with the Sense 10. The POs assessed were more accurate in SpO_2_ measurement when simulated perfusion levels were high.
